# Nitric oxide-mediated intersegmental modulation of cycle frequency in the crayfish swimmeret system

**DOI:** 10.1242/bio.032789

**Published:** 2018-05-01

**Authors:** Misaki Yoshida, Toshiki Nagayama, Philip Newland

**Affiliations:** 1 Division of Biology, Graduate School of Science and Engineering, Yamagata University, 990-8560, Yamagata, Japan; 2 Department of Biology, Faculty of Science, Yamagata University, 990-8560, Yamagata, Japan; 3 Center of Biological Sciences, University of Southampton, Highfield Campus, Southampton SO17 1BJ, UK

**Keywords:** Central pattern generator, Swimmeret rhythm, NO, Intersegmental coordination

## Abstract

Crayfish swimmerets are paired appendages located on the ventral side of each abdominal segment that show rhythmic beating during forward swimming produced by central pattern generators in most abdominal segments. For animals with multiple body segments and limbs, intersegmental coordination of central pattern generators in each segment is crucial for the production of effective movements. Here we develop a novel pharmacological approach to analyse intersegmental modulation of swimmeret rhythm by selectively elevating nitric oxide levels and reducing them with pharmacological agents, in specific ganglia. Bath application of L-arginine, the substrate NO synthesis, increased the cyclical spike responses of the power-stroke motor neurons. By contrast the NOS inhibitor, L-NAME decreased them. To determine the role of the different local centres in producing and controlling the swimmeret rhythm, these two drugs were applied locally to two separate ganglia following bath application of carbachol. Results revealed that there was both ascending and descending intersegmental modulation of cycle frequency of the swimmeret rhythm in the abdominal ganglia and that synchrony of cyclical activity between segments of segments was maintained. We also found that there were gradients in the strength effectiveness in modulation, that ascending modulation of the swimmeret rhythm was stronger than descending modulation.

## Abstract (precis)

**Summary:** We develop a novel pharmacological approach using a nitric oxide donor and a nitric oxide synthase inhibitor to analyse modulation and segmental synchrony in the swimmeret rhythm of the crayfish.

## INTRODUCTION

Central pattern generators (CPGs) are essential neural elements for the organization and patterning of locomotory motor output formation. In the early 1960 s, Hughes and Wiersma (1960) and Wilson (1961) showed that rhythmic motor patterns in arthropods were formed by an animal's central nervous system in the absence of sensory feedback. It is now well established that the walking and swimming movements of vertebrates and invertebrates are produced by underlying CPGs (Delcomyn, 1980; Abelew et al., 2000; Guertin, 2009). Furthermore, for animals with multiple body segments and limbs, intersegmental coordination of CPGs in local centres is essential for effective locomotion (Fuchs et al., 2011; Lambert et al., 2012; Tóth et al., 2012; Zhang et al., 2014; Couzin-Fuchs et al., 2015).

The crayfish, subphylum Crustacea, order Decapoda, swimmeret system has been studied extensively to understand the neural mechanism underlying intersegmental coordination. The swimmerets, or pleopods, are paired appendages located on the ventral side of each abdominal segment. Three pairs of swimmerets on the third to fifth abdominal segments in males, and four pairs from the second to fifth abdominal segments in females, beat rhythmically to generate forward thrust through cycles of power-stroke and return-stroke movements. They show rhythmic beating activity during egg ventilation, righting behaviour, forward swimming and walking.

The abdominal central nervous system comprises a chain of segmental ganglia, with each segmental pair of swimmerets being controlled by the corresponding segmental ganglion. The stroke of each swimmeret is controlled by a CPG activating antagonistic power- and return-stroke motor neurons that are active in strict anti-phase (Hughes and Wiersma, 1960). Rhythmic bursts of motor neuron spikes are generated by chains of serially repeated pairs of CPGs, one in each hemiganglion, that are interconnected both bilaterally across the midline and between abdominal segments (Ikeda and Wiersma, 1964; Wiersma and Ikeda, 1964). Mulloney and colleagues analysed the local pattern-generating circuit in the hemiganglion, and the descending and ascending pathways for intersegmental modulation of swimmeret rhythms [for reviews, see Mulloney and Smarandache (2010); Mulloney and Smarandache-Wellmann (2012)]. Each cycle of motor output begins with a burst of spikes in power-stroke motor neurons in the most posterior ganglion. Bursts of spikes in power-stroke motor neurons in more anterior ganglia follow their nearest posterior neighbour with a fixed phase lag. In each segment, bursts of spikes of left and right power-stroke motor neurons occur simultaneously. In each ganglion, nonspiking local interneurons determine when the power-stroke and return-stroke motor neurons are active (Smarandache-Wellmann et al., 2013). Furthermore, ascending and descending interneurons (Namba and Mulloney, 1999) coordinate neighbouring segmental CPGs by conducting information relating to phase to the local nonspiking commissural interneuron 1 (ComI1) in neighbouring anterior and posterior ganglia respectively (Mulloney and Hall, 2003) that are intercalated between the intersegmental interneurons and the CPG core. The ComI1 interneurons are key interneurons that coordinate intersegmental swimmeret beating (Mulloney et al., 2006; Mulloney and Hall, 2007). Changes in their membrane potential, due to synaptic inputs from ascending and descending intersegmental interneurons, modify the strength and phase of bursts of the rhythm, but do not affect the cycle frequency of the beating rhythm across segments (Smarandache-Wellmann et al., 2014). It is still unclear what mechanism underlies the modulation of swimmeret cycle frequency across multiple body segments.

The free radical nitric oxide (NO) is well known to modulate rhythmic motor activity induced by CPGs (Newland and Yates, 2007). NO inhibits the swimming rhythm of *Xenopus larvis* tadpoles (McLean and Sillar, 2002) and potentiates locomotor activity in the lamprey (Kyriakatos et al., 2009). NO further modulates swimming speed in the larval zebrafish (Severi et al., 2014) and walking speed in rats (Wang et al., 2001). In the swimmeret system of the crayfish, the co-application of L-arginine, the substrate for NO synthesis with a cholinergic agonist, carbamoylcholine chloride (carbachol), increases the cycle frequency of the power-stroke motor neurons, while the co-application of the nitric oxide synthase (NOS) inhibitor, N^G^-nitro-Larginine methyl ester (L-NAME) with carbachol decreases it (Mita et al., 2014). Here, we have analysed intersegmental modulation and changes in burst frequency in individual ganglia, and between ganglia, using a novel pharmacological approach using sequential local application of L-arginine and L-NAME to separate ganglia.

## RESULTS

### Central pattern generators in each abdominal ganglion

**Fig. 1.**
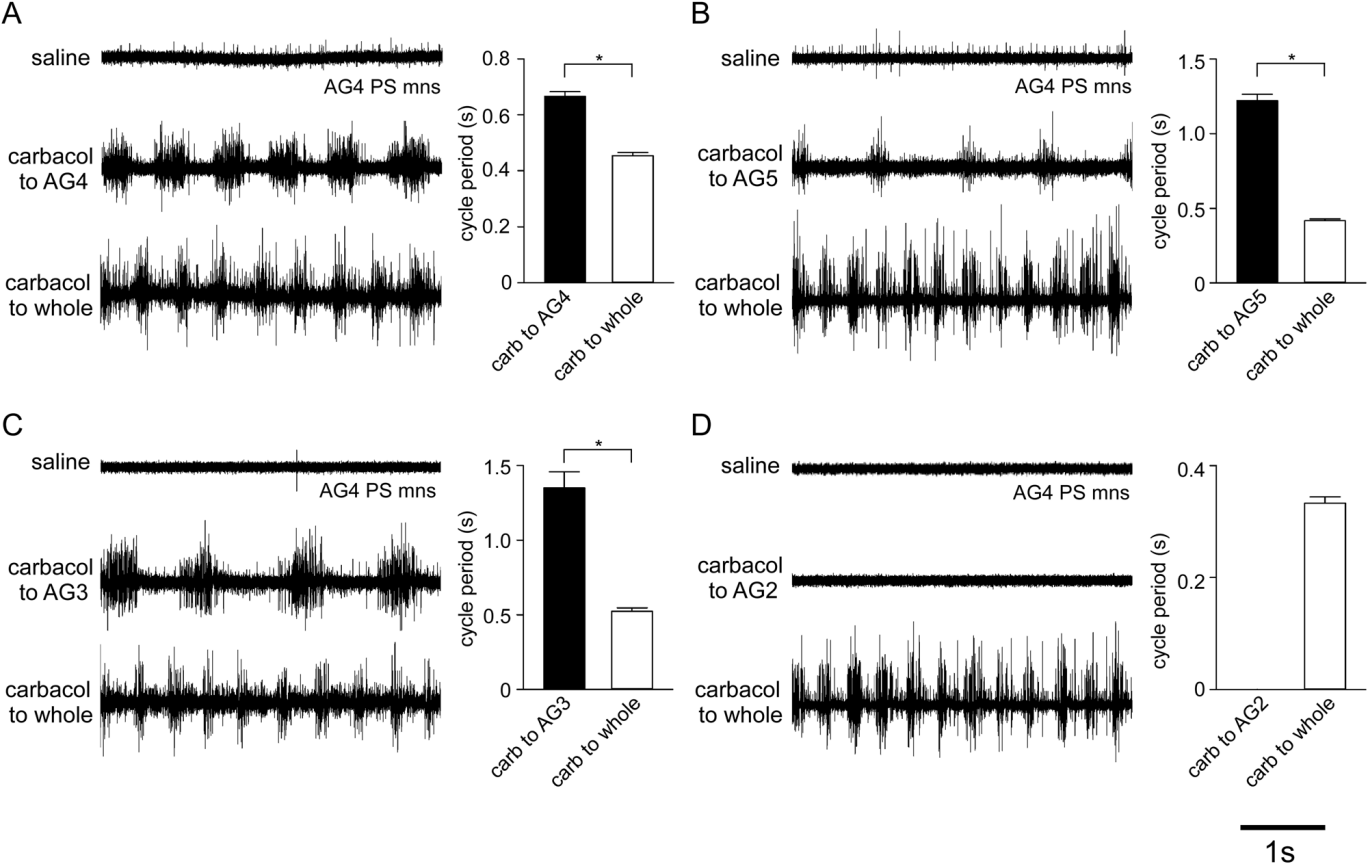
**Effect of local application of 8 μM carbachol on abdominal ganglia.** In each preparation, the spike activity of PS motor neurons in the fourth abdominal ganglion (AG4) was monitored with extracellular electrodes. Before application of carbachol, PS motor neurons were silent or spiked tonically at low frequencies in all preparations (top traces in A–D). Application of carbachol locally to AG4 (middle trace in A), AG5 (middle trace in B) or AG3 (middle trace in C) evoked rhythmic bursts of spikes in AG4 PS motor neurons. By contrast, local application of carbachol to AG2 caused no change in the activity of the AG4 PS motor neurons (middle trace in D). The cycle frequency of motor neuron activity increased after exchanging the external bathing solution from normal to carbachol-containing saline (bottom traces in A–D).

In isolated abdominal nerve cord preparations, swimmeret motor neurons showed either no spontaneous spike activity or tonic spike activity at a low frequency. For example, AG4 power-stroke (PS) motor neurons showed no rhythmic bursts before 8 μM carbachol application (top trace in Fig. 1A–D). When carbachol-containing saline was applied within the petroleum-jelly well surrounding AG4, PS motor neurons of the same ganglion showed rhythmic bursts of spikes with a frequency of 0.66±0.02 s (*n*=7, mean±s.e.m.) (middle trace in Fig. 1A). Similarly, local application of carbachol within the petroleum-jelly well around AG5 or AG3 [0.98±0.03 s (*n*=4) and 1.08±0.09 s (*n*=3), respectively] evoked rhythmic bursts of motor neuron spikes in AG4 PS motor neurons (middle traces in Fig. 1B,C). Local application of carbachol to a single ganglion therefore activated the CPG circuit within that ganglion, but also revealed the presence of both ascending and descending pathways to drive CPGs in adjacent ganglia.

The cycle period of the PS motor neurons was longer when the CPG of a single ganglia was activated compared to carbachol application to the entire abdominal nerve cord. For example, when carbachol was additionally applied to the remaining ganglia, by exchanging the external bathing solution in the experimental chamber from normal saline to carbachol-containing saline, the cycle frequency of the burst of motor neuron spikes significantly increased to 0.38±0.03 s (mean±s.e.m., *n*=4) (bottom traces in Fig. 1A–C) for each preparation (Student’s *t*-tests, *P*>0.05). Local application of carbachol to a well around AG2 failed to elicit rhythmic activity in AG4 PS motor neurons, although carbachol applied to the bathing solution surrounding the remaining ganglia caused burst of spikes in AG4 PS motor neuron with a cycle period of 0.33±0.01 s (*n*=6) (middle and bottom traces in Fig. 1D).

**Fig. 2.**
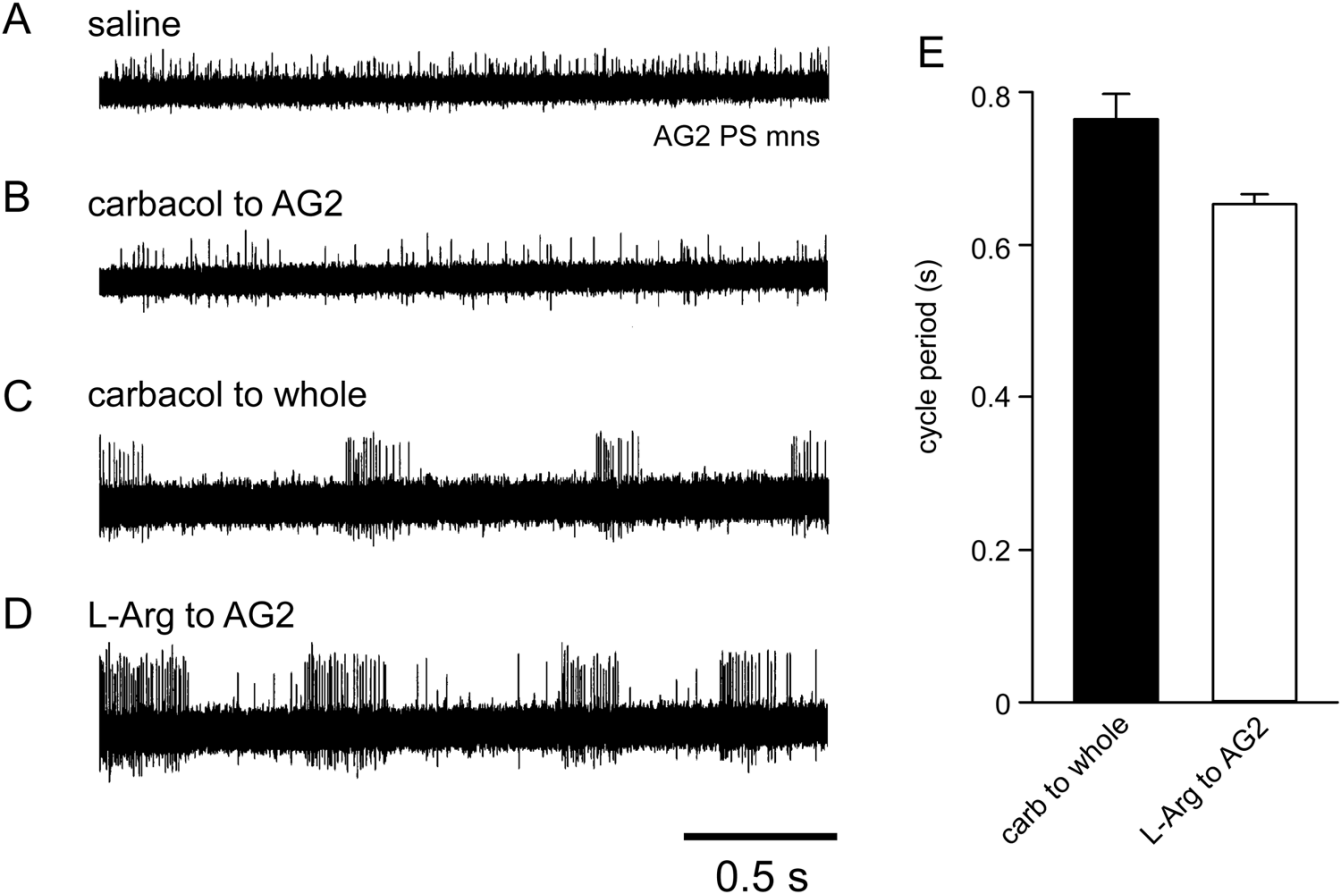
**CPG in AG2.** Extracellular recordings of the spike activity of PS motor neurons in AG2 before (A) and after (B) local carbachol application to AG2. The AG2 PS motor neurons showed a continuous discharge of tonic spikes. Replacing the external bathing solution to carbachol-containing saline evoked rhythmic bursts of spikes of AG2 PS motor neurons (C) whose cycle frequency increased after local application of L-arginine to AG2 (D).

Direct local application of carbachol to AG2 (*n*=3) did not evoke any rhythmic activity in the AG2 PS motor neurons (Fig. 2A,B). By contrast, rhythmic bursts of spikes in AG2 PS motor neurons were observed on application of carbachol to the bathing solution surrounding the remaining ganglia with a cycle period of 0.77±0.03 (mean±s.e.m., *n*=3) (Fig. 2C,E). Subsequent local application of 5 mM L-arginine (L-arg) to the AG2 well caused a decrease in the cycle period of the swimmeret rhythm to 0.65±0.01 s (*n*=3) (Fig. 2D,E). These results suggest that while the AG2 CPG itself cannot elicit rhythmic activity of AG2 motor neurons*,* descending and ascending pathways from/to AG2 CPG exist to modulate cycle frequency in other segmental ganglia.

**Fig. 3.**
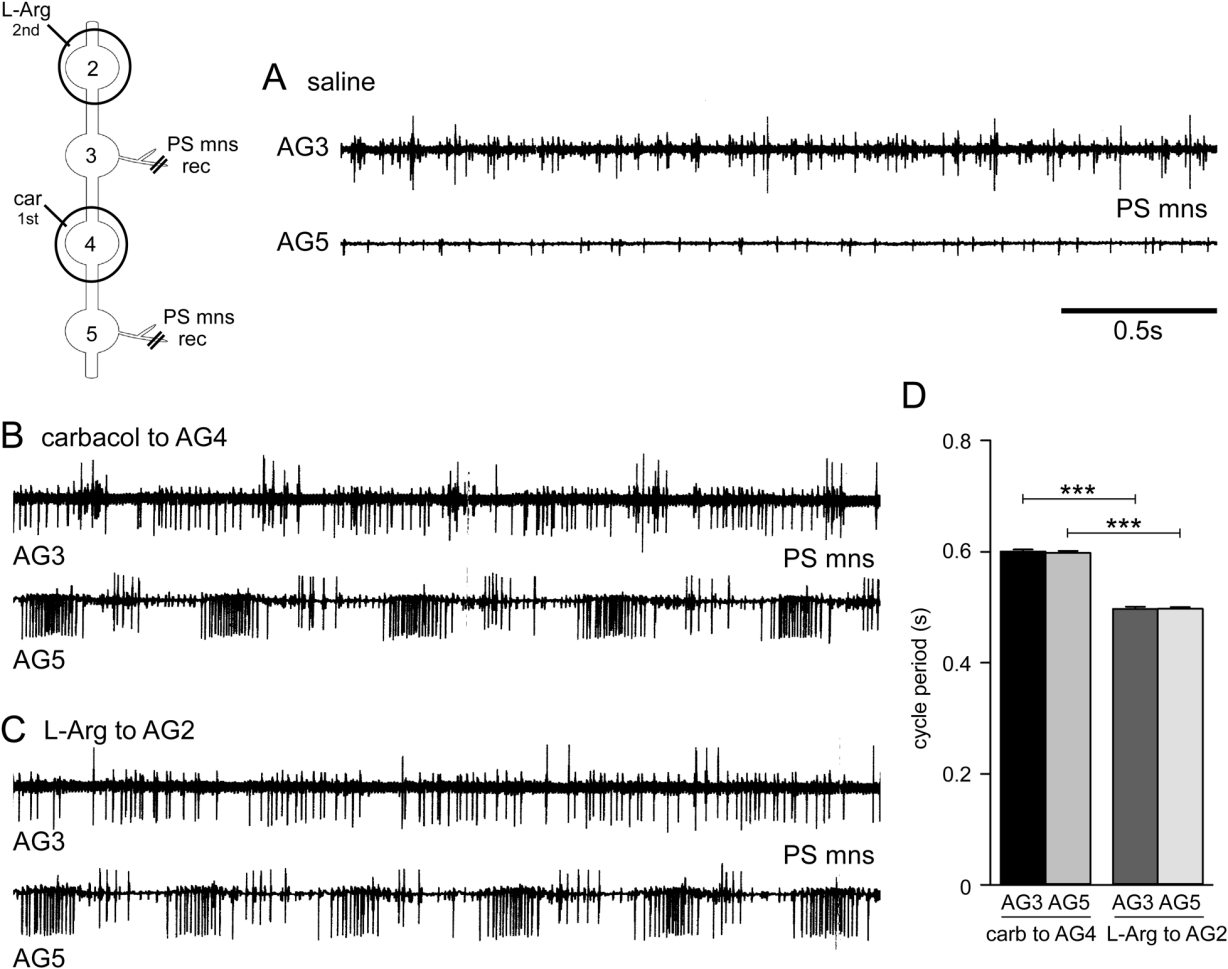
**Synchronization of CPGs between abdominal segments.** (A) Simultaneous recordings from PS motor neurons in AG3 and AG5 showed no rhythmic activity when bathed in saline. (B) Carbachol application to AG4 (see inset) evoked rhythmic motor activity in both sets of PS motor neurons. (C) Subsequent application of L-arginine to AG2 reduced the cycle period in both sets of motor neurons. (D) Both AG3 and AG4 PS motor neuron showed a statistically significant reduction in cycle period following L-arginine application to AG2 (one-way RM ANOVA, ***P<0.001). There were no statistically significant differences between PS motor neuron cycle periods during carbachol application to AG4, nor following application of L-arginine application to AG2.

To determine whether synchronization between the CPGs was maintained during L-arginine application the PS motor neurons of AG3 and AG5 were recorded simultaneously. The PS motor neurons showed no rhythmic activity when bathed in saline (Fig. 3A). The addition of carbachol to the well around AG4 evoked rhythmic motor activity in both sets of PS motor neurons with a cycle period of 0.600±0.04 s (*n*=20) in AG3 and 0.598±0.04 s in AG5 (Fig. 3B). There was no significant difference between the cycle periods of both sets of PS motor neurons following carbachol treatment [one-way repeated measures (RM) ANOVA, d.f.=19, *P*=0.851]. The subsequent application of L-arginine to AG2 reduced the cycle period in both sets of motor neurons to 0.497±0.04 s in AG3 and 0.498±0.04 s in AG5 (Fig. 3C). Statistical analysis revealed that the reduction in cycle period in AG3 and AG5 motor neurons was significant (one-way RM ANOVA, *P*<0.001 for AG3 and AG5). Following L-arginine application there was again no significant difference between the cycle periods of the AG3 and AG5 motor neurons (one-way RM ANOVA, *P*=0.920) (Fig. 3D).

### Ascending modulation of swimmeret beating

**Fig. 4.**
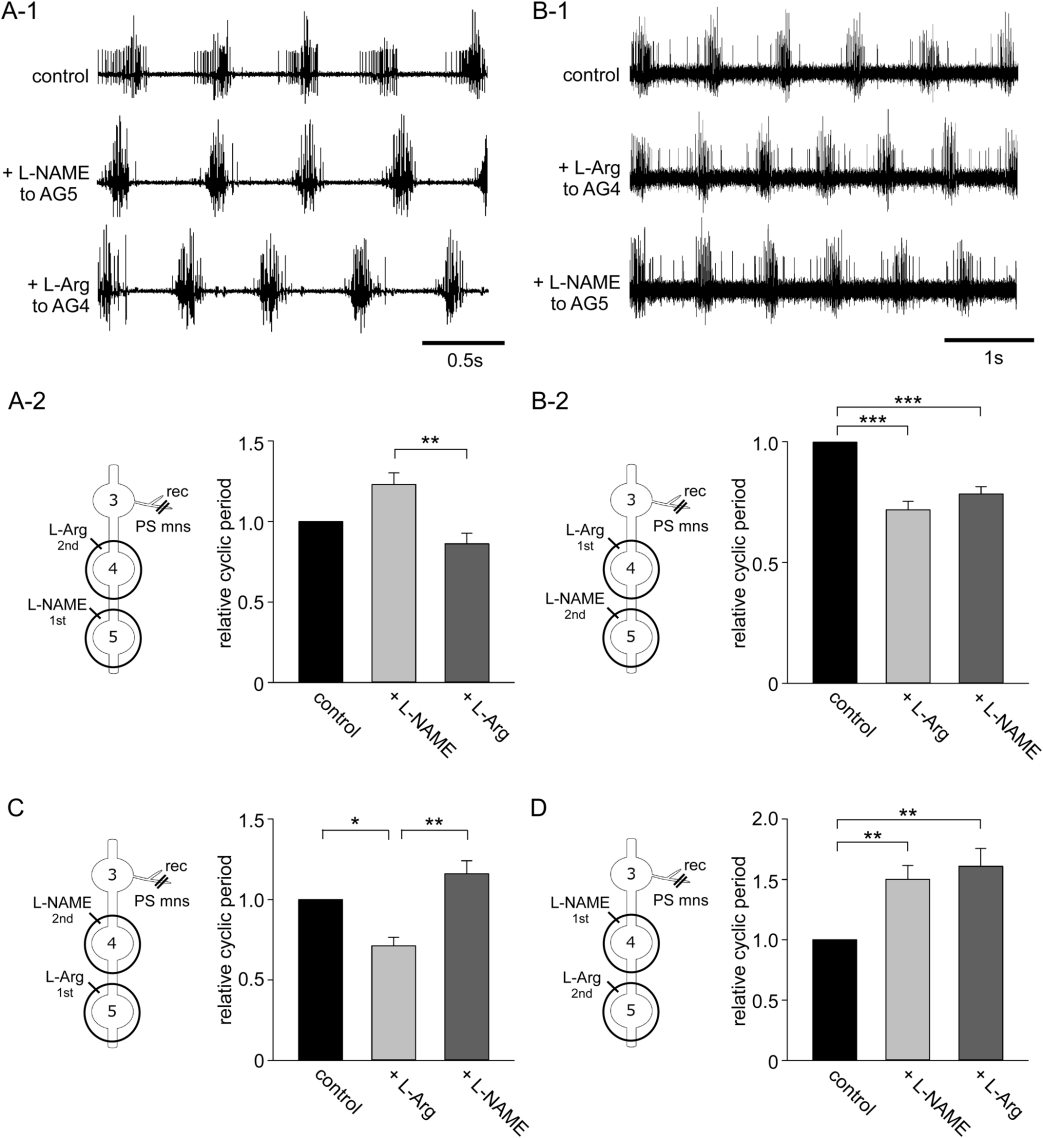
**Ascending modulation of swimmeret cycle frequency.** (A-1–2) Sequential local application of L-NAME to AG5 followed by L-arginine to AG4. (A-1, top) Application of carbachol to the external bathing solution evoked rhythmic activity in AG3 PS motor neurons. (Middle) Replacing the normal saline within the well around AG5 with L-NAME-containing saline increased the cycle period of the PS motor neurons. (Bottom) Replacing the normal saline within the well around AG4 with L-arginine-containing saline decreased the cycle period of the motor neurons. (A-2) Relative changes in the cycle period of rhythmic bursts of motor neuron spikes. Asterisks indicate significant differences (**P<0.01). (B-1–2) Sequential application of L-arginine to AG4 followed by L-NAME to AG5. (B-1, top) Carbachol in the external bathing solution evoked rhythmic activity in AG3 PS motor neurons. (Middle) Exchanging the normal saline within the AG4 well to L-arginine-containing saline decreased the relative cycle period of motor neuron activity. (Bottom) Exchanging the normal saline in the AG5 well to L-NAME-containing saline also decreased the cycle period. (B-2) Relative changes in the cycle period of rhythmic bursts of motor neuron spikes. (C) Relative changes in the cycle period of rhythmic bursts of motor neuron spikes after sequential application of L-arginine to AG5 followed by L-NAME to AG4. (D) Relative changes in the cycle period of rhythmic bursts of motor neuron spikes after sequential application of L-NAME to AG4 followed by L-arginine to AG5. Asterisks indicate significant differences (*P<0.05; **P<0.01; ***P<0.001). Schematic diagrams of experimental treatments are shown to the left.

Nitric oxide (NO) is known to modulate swimmeret beating in crayfish, and L-arginine and L-NAME have opposite effects on NO levels and have opposing effects on the cycle period of the swimmeret rhythm (Mita et al., 2014). L-NAME applied to a single ganglion increased the cycle period of the swimmeret rhythm (middle trace in Fig. 4A-1), while L-arginine decreased the cycle period (middle trace in Fig. 4B-1).

The modulatory effects of L-NAME and L-arginine on ascending modulation of the swimmeret rhythm were analysed by varying the order of application of the two drugs to different abdominal ganglia. Rhythmic activity of the PS motor neurones recorded from AG3 was induced by applying carbachol dissolved in normal saline to the main bathing solution (top trace in Fig. 4A-1). The cycle period of the PS motor neurons was 508.2±2.8 ms (mean±s.e. of the mean) in this preparation. When normal saline inside an AG5 well was replaced with 5 mM L-NAME-containing saline, the cycle period of the AG3 PS motor neurons increased to 589.1±3.2 ms (middle trace in Fig. 4A-1). A subsequent exchange of saline inside the AG4 well to 5 mM L-arginine shortened the cycle period to 522.9±3.4 ms (bottom trace in Fig. 4A-1). Application of L-NAME first to AG5 led to an increase in the cycle period of PS motor neurones compared to control (from 696.9±54.9 ms to 879.5±138.2 ms, *n*=6 for each) (Fig. 4A-2). Subsequent application of L-arginine to the ganglion nearest to the recording site, AG4, reversed the effects of L-NAME, leading to a decrease in cycle period of 579.8±40.7 ms. Thus, application of L-NAME to AG5 increased the cycle period to 1.23±0.07 while subsequent application of L-arginine to AG4 decreased it to 0.86±0.07. There was a significant difference in cycle period between L-NAME and L-arginine (*P*=0.008, d.f.=5, one-way RM ANOVA). Next, the order of presentation of the drugs to the ganglia was reversed (Fig. 4B-2) so that L-arginine was applied to AG4 first, and then L-NAME was applied to AG5. When the bathing solution was exchanged from normal saline to carbachol-including saline, the cycle period of swimmeret beating activity of AG3 PS motor neurons was 894.0±7.8 ms in this preparation (top trace in Fig. 4B-1). Local application of L-arginine to the AG4 well decreased the cycle period to 751.7±7.8 ms (middle trace in Fig. 4B–1). The subsequent application of L-NAME to the AG5 had little effect on the cycle period of 758.7±12.9 ms (bottom trace in Fig. 4B–1). Thus, L-arginine applied to AG4 decreased the relative cycle period to 0.72±0.04 (*n*=5), while subsequent application of L-NAME to AG5 had little effect (0.78±0.03) (Fig. 4B–2). There were significant differences between control and L-arginine treatments (*P*<0.001, d.f.=4, one-way RM ANOVA) and between control and L-NAME treatments (*P*<0.001, d.f.=4, one-way RM ANOVA), although there were no statistically significant differences between L-arginine and L-NAME treatments (*P*=0.081; one-way RM ANOVA).

A similar pattern of modulation was found when the order of application of drugs was reversed. L-arginine applied first to AG5 led to a decrease in cycle period compared to control (1139.9±44.3 ms and 809.1±55.7 ms, respectively; *n*=5) (Fig. 4C). Subsequent application of L-NAME to the nearest ganglion to the recording site, AG4, reversed the effects of L-arginine application, leading to an increase in cycle period (1322.6±106.1 ms). Thus L-arginine decreased the relative cycle period to 0.71±0.05, while subsequent application of L-NAME increased the relative cycle period to 1.16±0.08. There were significant differences between control and L-arginine treatments (*P*=0.013, d.f.=4, one-way RM ANOVA) and between L-NAME and L-arginine treatments (*P*=0.001, d.f.=4, one-way RM ANOVA). By contrast, when L-NAME was applied first to AG4 there was again an increase in cycle period (from 793.4±100.8 ms to 1159.5±106.0 ms; *n*=5), but subsequent application of L-arginine to the more distal AG5 had no significant effect on the cycle period (1222.6±81.5 ms) (Fig. 4D). Thus, L-NAME applied to AG4 increased the relative cycle period to 1.50±0.11, while subsequent application of L-arginine to AG5 had little effect (1.61±0.15). There were significant differences between control and L-NAME treatments (*P*=0.005, d.f.=4, one-way RM ANOVA) and between control and L-arginine treatments (*P*=0.002, d.f.=4, one-way RM ANOVA), however, there was no statistical difference between L-NAME and L-arginine treatments (*P*=0.384, d.f.=4, one-way RM ANOVA).

Taken together these results show that the effect of drugs applied initially to more distant ganglia from the recording site were negated or reversed by drugs that had the opposing effect applied subsequently to nearer ganglion. Thus, ascending modulation from the nearest most posterior ganglion is stronger than that from more distal ganglia.

### Descending modulation of swimmeret beating

The modulatory effects of L-NAME and L-arginine on descending modulation of the swimmeret rhythm was also analysed by varying the order of application of the two drugs to different abdominal ganglia. Results showed that that descending modulation from nearest anterior ganglion to the recording site was stronger than that from more distal anterior ganglion.

**Fig. 5.**
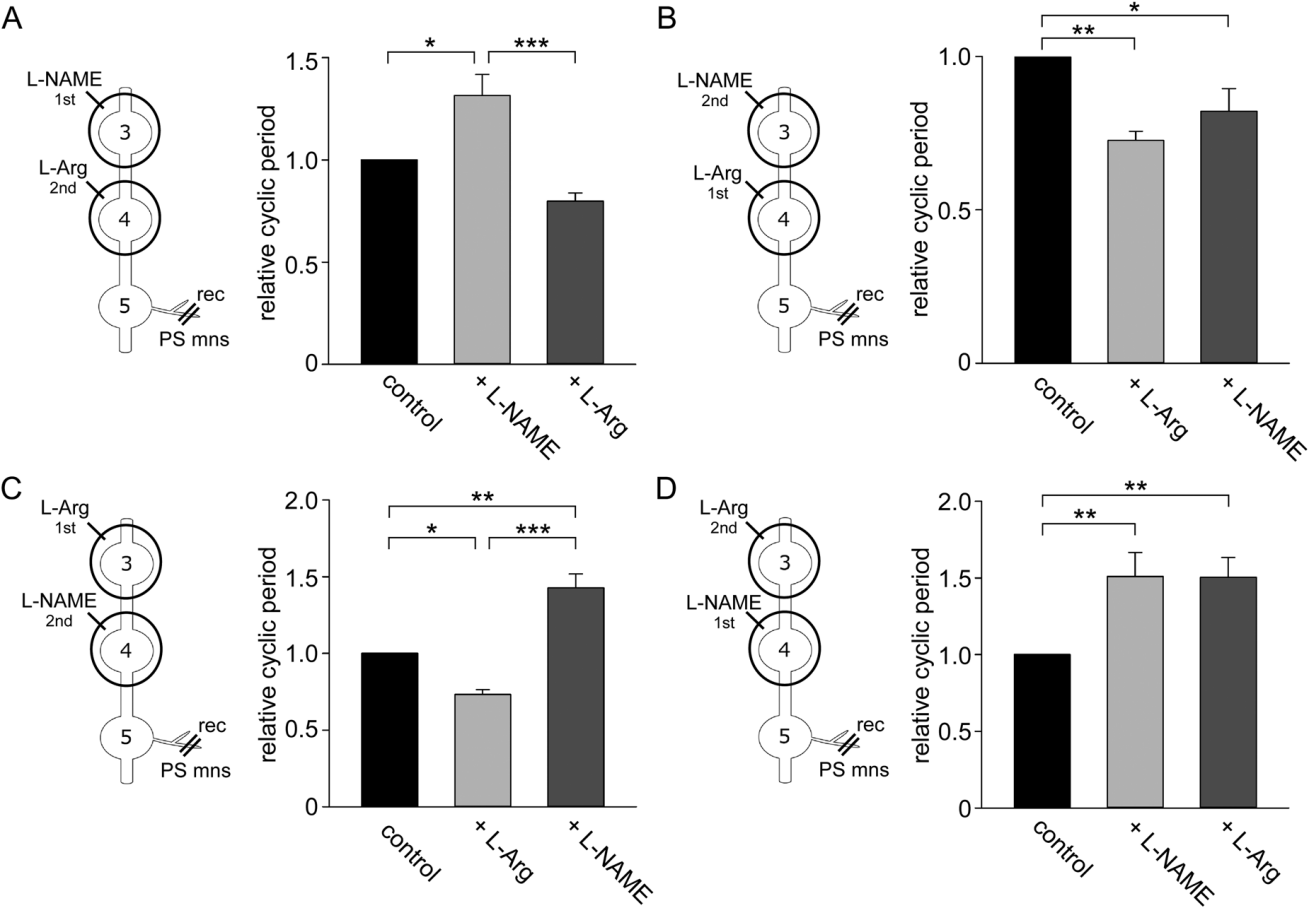
**Descending modulation of swimmeret cycle frequency.** (A) Relative changes in the cycle period of rhythmic bursts of motor neuron spikes recorded from AG5 after sequential application of L-NAME to AG3 followed by L-arginine to AG4. (B) Relative changes in the cycle period of rhythmic bursts of motor neuron spikes after sequential application of L-arginine to AG4 followed by L-NAME to AG3. (C) Relative changes in the cycle period of rhythmic bursts of motor neuron spikes after sequential application of L-arginine to AG3 followed by L-NAME to AG4. (D) Relative changes in the cycle period of rhythmic bursts of motor neuron spikes after sequential application of L-NAME to AG4 followed by L-arginine to AG3. Asterisks indicate significant differences (*P<0.05; **P<0.01; ***P<0.001). Schematic diagrams of experimental treatments are shown to the left.

Application of L-NAME first to AG3 led to an increase in the cycle period of the rhythm recorded from PS motor neurones recorded from AG5 compared to control (979.5±67.7 ms and 1283.2±115.8 ms, respectively, *n*=7 for both treatments) (Fig. 5A). Subsequent application of L-arginine to the nearest ganglion to the recording site, AG4, reversed the effects of L-NAME application, leading to a decrease in cycle period (777.4±57.7 ms). Thus L-NAME applied to AG3 increased the relative cycle period to 1.31±0.10, while L-arginine applied to AG4 shortened it to 0.80±0.04. There were significant differences between control and L-NAME treatments (*P*=0.016, d.f.=6, one-way RM ANOVA) and between L-NAME and L-arginine treatments (*P*<0.001, d.f.=6, one-way RM ANOVA).

By contrast, when L-arginine was applied first to AG4 there was a decrease in cycle period compared to control (699.8±102.1 ms and 509.4±83.2 ms, respectively, *n*=6 for each treatment) (Fig. 5B) but this effect was not reversed by application of L-NAME to the more anterior AG3 (590.6±134.2 ms). Local application of L-arginine to AG4 shortened the relative cycle period to 0.73±0.03 while subsequent application of L-NAME to AG3 caused little change, 0.82±0.07. There were significant differences between control and L-arginine treatments (*P*=0.002, d.f.=5, one-way RM ANOVA) and between control and L-NAME treatments (*P*=0.018, d.f.=5, one-way RM ANOVA), although there was no statistically significant difference between L-arginine and L-NAME treatments (*P*=0.113, d.f.=5, one-way RM ANOVA).

A similar pattern of modulation was found when the application of drugs was reversed. Application of L-arginine first to the anterior AG3 led to a decrease in the cycle period compared to control (748.6±124.9 ms and 558.5±114.4 ms, respectively, *n*=5) (Fig. 5C). Subsequent application of L-NAME to the nearest ganglion to the recording site, AG4, led to an increase in the cycle period (1067.3±186.2 ms), thereby reversing the effects of the L-arginine. Local application of L-arginine to AG3 shortened the relative cycle period to 0.73±0.03 while subsequent local application of L-NAME to AG4 increased it to 1.43±0.09. There were statistically significant differences between control and L-arginine treatments (*P*=0.011, d,f,=4, one-way RM ANOVA) between control and L-NAME (*P*=0.002, d.f.=4, one-way RM ANOVA) treatments, and between L-arginine and L-NAME treatments (*P*<0.001, d.f.=4, one-way RM ANOVA). By contrast, when L-NAME was applied first to AG4 there was again an increase in cycle period from 865.6±131.4 ms to 1225.0±144.3 ms (*n*=6) (Fig. 5D) but subsequent application of L-arginine to the more anterior AG3 had no effect on the cycle period (1259.3±193.1 ms). Thus L-NAME applied to AG4 increased the relative cycle period to 1.51±0.16, while subsequent application of L-arginine to AG3 had little effect on the cycle period (1.51±0.13). There were statistically significant differences between control and L-NAME treatments (*P*=0.008, d.f.=5, one-way RM ANOVA) and between control and L-arginine treatments (*P*=0.006, d.f.=5, one-way RM ANOVA). There was, however, no statistically significant difference between L-NAME and L-arginine treatments (*P*=0.974, d.f.=5, one-way RM ANOVA).

Together these results indicate that descending modulation from the nearest anterior ganglion to the recording site of the PS motor neurons was stronger than that from more distal anterior ganglion.

### Different strengths of ascending and descending modulation

**Fig. 6.**
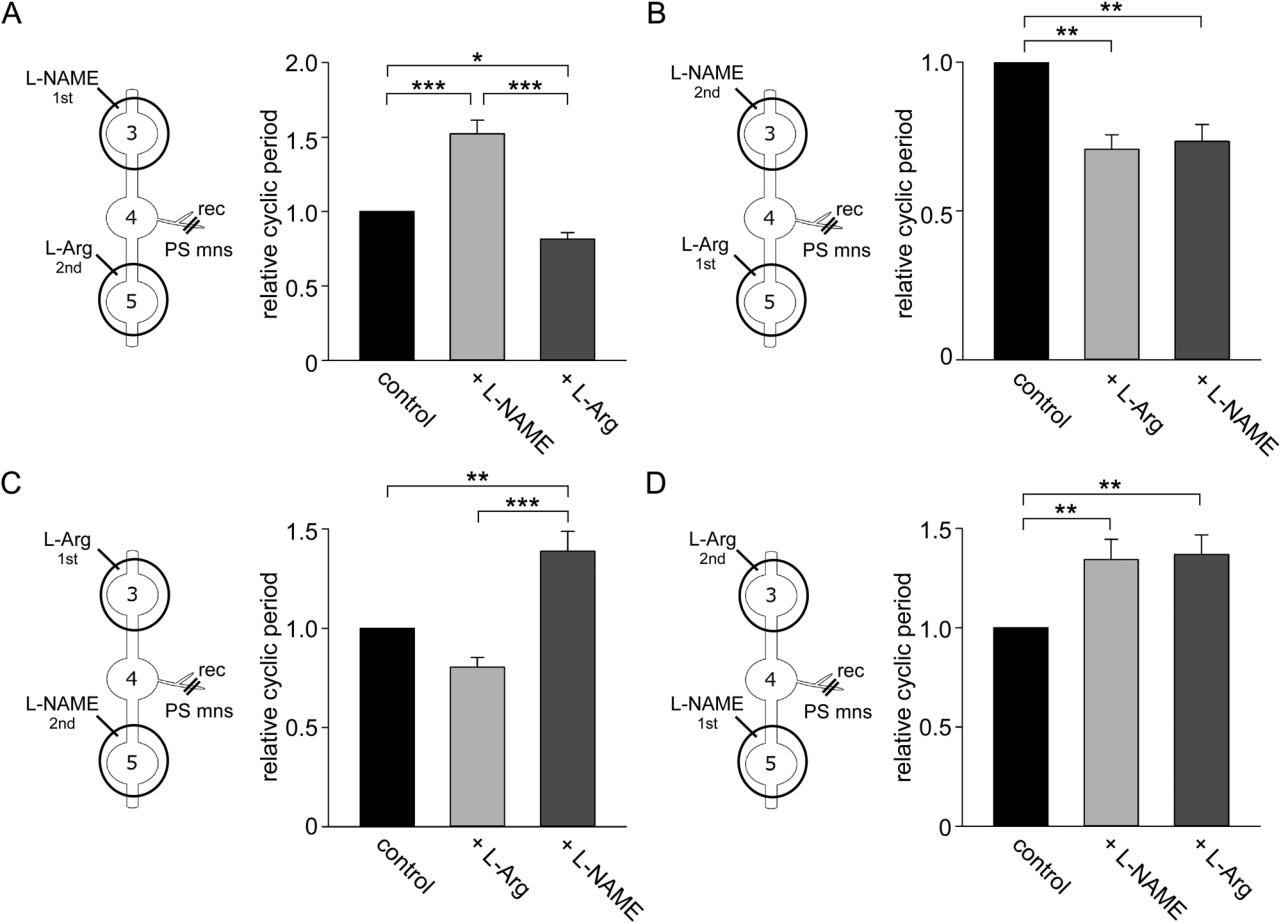
**Descending and ascending modulation of swimmeret cycle frequency.** (A) Relative changes in the cycle period of rhythmic bursts of motor neuron spikes recorded from AG4 after sequential application of L-NAME to AG3 followed by L-arginine to AG5. (B) Relative changes in the cycle period of rhythmic bursts of motor neuron spikes after sequential application of L-arginine to AG5 followed by L-NAME to AG3. (C) Relative changes in the cycle period of rhythmic bursts of motor neuron spikes after sequential application of L-arginine to AG3 followed by L-NAME to AG5. (D) Relative changes in the cycle period of rhythmic bursts of motor neuron spikes after sequential application of L-NAME to AG5 followed by L-arginine to AG3. Asterisks indicate significant differences (*P<0.05; **P<0.01; ***P<0.001). Schematic diagrams of experimental treatments are shown to the left.

To compare the strength of ascending and descending modulation, the spike activity of AG4 PS motor neurons was monitored and drugs applied anteriorly to AG3 and posteriorly to AG5 (Fig. 6).

Application of L-NAME to the anterior AG3 (Fig. 6A) increased the length of the cycle period compared to control (1053.0±85.4 ms and 1614.4±205.6 ms, respectively; *n*=5). The subsequent application of L-arginine to the posterior AG5 reduced the cycle period to 855.2±79.8 ms thereby reversing the initial L-NAME effect. Thus L-NAME to AG3 increased the relative cycle period to 1.52±0.09 while subsequent application of L-arginine to AG5 shortened the relative cycle period to 0.81±0.04. There were statistically significant differences between L-arginine and L-NAME treatments (*P*<0.001, d.f.=4, one-way RM ANOVA), between control and L-NAME treatments (*P*<0.001, d.f.=4, one-way RM ANOVA) and between control and L-arginine treatments (*P*=0.035, d.f.=4, one-way RM ANOVA).

Application of L-arginine first to AG5 reduced the cycle period compared to control (1075.6±103.4 ms and 761.1±95.0 ms, respectively; *n*=5) (Fig. 6B). Subsequent application of L-NAME to AG3 caused little change in the cycle period of 783.8±92.6 ms. Thus, the application of L-arginine to AG5 shortened the relative cycle period to 0.71±0.05 while subsequent application of L-NAME to AG3 had little effect on the cycle period of  0.73±0.06. There were statistically significant differences between control and L-arginine treatments (*P*=0.001, d.f.=4, one-way RM ANOVA) and between control and L-NAME treatments (*P*=0.001, d.f.=4, one-way RM ANOVA), but no statistically significant difference between L-arginine and L-NAME treatments (*P*=0.609, d.f.=4, one-way RM ANOVA).

These two experiments show that irrespective of what drug is applied to AG3 the dominant effect depended on the application of the drug to the posterior AG5, which implies that ascending modulation was more dominant than descending modulation. This was confirmed by exchanging the drugs applied to the anterior and posterior ganglia and their order of application. L-arginine applied first to AG3 reduced the cycle period compared to control (1013.5±79.3 ms and 823.0±98.2 ms, respectively, *n*=6) (Fig. 6C). The subsequent application of L-NAME to the posterior AG5 increased the cycle period to 1404.8±133.2 ms thereby reversing the effect of the initial application of L-arginine to the anterior ganglion. Thus, the application of L-arginine to AG3 shortened the relative cycle period to 0.80±0.05 while the subsequent application of L-NAME to AG5 increased the relative cycle period to 1.39±0.10. There was a statistically significant difference between control and L-NAME treatments (*P*=0.005, d.f.=5, one-way RM ANOVA), and between L-arginine and L-NAME treatments (*P*<0.001, d.f.=5, one-way RM ANOVA).

Furthermore, L-NAME applied first to AG5 increased the cycle period compared to control (938.7±171.1 ms and 1241.0±214.7 ms, respectively, *n*=5) (Fig. 6D). The subsequent local application of L-arginine to AG3 had little effect on the cycle period (1247.8±193.3 ms). Thus, application of L-NAME to AG5 increased the relative cycle period to 1.34±0.10 while subsequent application of L-arginine to AG3 had little effect on the relative cycle period of 1.37±0.10. There were statistically significant differences between control and L-NAME treatments (*P*=0.008, d.f.=4, one-way RM ANOVA) and between control and L-arginine treatments (*P*=0.008, d.f.=4, one-way RM ANOVA) but not between L-Name and L-arginine treatments (*P*=0.764, d.f.=4, one-way RM ANOVA<0.001) These results again showed that ascending modulation was more predominant than descending modulation.

## DISCUSSION

Despite many studies on the control of swimmeret beating over recent decades the results of this study reveal that the cholinergic agonist, carbachol, does not evoke rhythmic activity of the swimmerets when applied to the second abdominal ganglion. The implication of this is that the AG2 does not contain a CPG that can initiate rhythmic activity in other ganglion. We have also developed a novel method using NO to unpick the modulatory effects of a rhythmic system and show that there are gradients in the effect of modulation. We show that nitric oxide has the potential to modulate and synchronize the CPGs in other ganglion and that its effect is likely to occur through ascending and descending interneurons that lie outside the segmental CPGs.

### CPGs in each abdominal ganglion

We have shown that the activation of local CPGs, from AG3–AG5, elicited rhythmic bursts of motor neuron spikes in posterior and/or anterior ganglia. The AG3–AG5 CPGs are thus interconnected by ascending and/or descending intersegmental interneurons and have the ability to generate rhythmic burst activity of the swimmeret motor neurons of all abdominal segments. As Acevedo et al. (1994) and Braun and Mulloney (1993) have indicated, the cycle frequency of the power stroke (PS) motor neurons was slower when a single ganglion was activated by local application of carbachol compared to the cycle period of the rhythm when the entire nervous system was bathed in carbachol. We also found that the local application of carbachol around AG2 did not elicit rhythmic activity of the motor neurons in either AG2 itself or posterior ganglia, although the cycle frequency elicited by carbachol applied in the bathing solution was modulated by local application of L-arginine on AG2. Acevedo et al. (1994) showed that proctolin applied only to AG2 elicits swimmeret activity in more posterior ganglia in only two of four cases. Thus, the CPG in AG2 appears to have a very weak influence on rhythmic patterning of the PS motor neurons, which differs markedly from the effects caused by CPGs in all other abdominal ganglia. This difference may well reflect the different roles of the swimmerets on the different abdominal segments, where those on the second abdominal segment in males lost their function to propel the animal through the water during evolution.

Despite a lack of CPG activity in AG2 the effects of L-arginine application on AG2 changed the cycle frequency in more posterior ganglia, with synchrony between the ganglia being maintained. Given the modulatory action of L-arginine without concomitant CPG activity this implies that intersegmental interneurons outside the CPG are involved in mediating the modulatory effects. We know from other studies that NO modulates reflex activity in the terminal abdominal ganglion of crayfish (Araki et al., 2004), and that many intersegmental interneurons in the terminal ganglion with ascending axons are modulated by NO (Aonuma and Newland, 2001). Moreover, Schuppe et al. (2001) showed that a number of the ascending interneurons in the terminal ganglion that process mechanosensory signals are likely to contain NOS. It seems reasonable to hypothesize, therefore, that the effects of NO in the swimmeret system are acting on intersegmental interneurons that convey information between segmental CPGs rather than on the CPGs themselves.

### Gradient effects of ascending and descending modulation

In terms of the ascending and descending intersegmental modulation we found in this study, the cycle frequency of motor neuron bursts was dependent on the ganglion that the drug was applied to. The effect of a drug applied initially to a more distant ganglion was negated and/or reversed by a drug that had the opposite effect on NO levels applied subsequently to nearer ganglion. Thus, the influence of activity in the nearest neighbour ganglion had a dominant effect on the PS motor neurons of a given ganglion. Moreover, the effects of application of L-arginine and L-NAME to more distant ganglia were not observed when drugs with opposing effect, L-NAME and L-arginine, respectively, were first applied to nearer ganglia. Both ascending and descending modulation of the swimmeret cycle period therefore showed gradients in strength along the abdominal nervous system. Furthermore, ascending information of cycle period was stronger than descending modulation. The effects of sequential application of drugs to neighbouring anterior ganglion were reversed when different drugs were applied first to neighbouring posterior ganglia. Our results therefore show parallels with those of Mulloney et al. (2006) who found segmental gradients in the responsiveness of coordinating interneurons in different abdominal segments. The intersegmental modulation we describe here contrasts with what we know in some other arthropod control systems. For example, Ludwar et al. (2005) showed that when rhythmic activity was activated in one thoracic ganglion in the stick insect, *Carausius morosus*, it had little effect on the spontaneous activity of leg motor neurons in a neighbouring ganglion even though ganglia showed coordinated activity. Similarly, the ascending and descending modulation of rhythmic activity we found in crayfish also contrasts with a lack of an intersegmental influence in leg motor patterns in isolated locust thoracic nerve cords (Ryckebusch and Laurent, 1994), suggesting that not all CPGs underlying coordinated movements of multiple appendages across many body segments share similar underlying control mechanisms.

## MATERIALS AND METHODS

Female crayfish *Procambarus clarkii* Girard, 5–9 cm body length from rostrum to telson were used in all experiments. They were obtained locally from a commercial supplier (Okayama, Japan), maintained in freshwater laboratory tanks at room temperature (23–25°C) and fed weekly on a diet of chopped potato and liver.

### Preparations and extracellular recording

The abdominal nerve chain from the first to sixth (terminal) abdominal ganglion with relevant nerve roots was isolated from the abdomen and pinned, dorsal side up in a 4 ml Sylgard-lined chamber containing physiological saline (van Harreveld, 1936). The dorsal ganglionic sheaths, from the second to the fifth abdominal ganglion, were surgically removed with fine forceps to facilitate drug perfusion. The spike activity of swimmeret PS motor neurons was recorded extracellularly using suction electrodes on the posterior branch of the first motor root in either the second, third, fourth or fifth abdominal ganglia.

### Pharmacological agents

The following pharmacological agents were obtained from Sigma-Aldrich: carbachol used as a cholinergic agonist, L-arginine as a substrate for endogenous NO synthesis and L-NAME as a NOS inhibitor. The concentrations of drugs used in this study were based on those used by Mita et al. (2014). The drugs were dissolved in physiological saline to 8 μM concentration of carbachol, 5 mM concentration of L-arginine and 5 mM concentration of L-NAME and were freshly prepared prior to application and used within 5 min.

### Preparations for drug application

A petroleum jelly and liquid paraffin (3:1 mixture) circular well was constructed around an individual ganglion, or multiple ganglia, under a binocular wide-field dissecting microscope. The well isolated individual ganglia from the bathing solution around the remaining parts of the nerve chain in the experimental chamber and was filled with a drop of physiological saline. Subsequently an extracellular suction electrode was placed on the nerve root and the preparation allowed to rest for 10 min. In these isolated preparations the swimmeret motor neurons were usually silent or showed tonic spikes at a low frequency.

For the first series of experiments, in which three to five animals were tested under each experimental condition (Figs 1–3), the bathing solution inside a well, surrounding one or more of AG2–AG5, was removed using filter paper and replaced with a drop of 8 μM carbachol-containing saline. PS motor neurons were recorded in one or more of AG2–AG5. This procedure was then repeated a further two times to ensure the carbachol was not diluted by any remaining liquid in the well. The spike activity of the motor neurons was recorded 10 min after the third replacement of the carbachol-containing saline for 5 min. Following this, the bathing solution of the normal saline outside the well, or wells, was washed out three times with carbachol-containing saline. Synchronization of effects of L-arginine application was tested by recording the PS motor neurons of AG3 and AG5 simultaneously, while applying carbachol to a well around AG4, and L-arginine to a well around AG2.

For the second series of experiments (Fig. 4), petroleum-jelly wells were constructed around each of the fourth (AG4) and fifth (AG5) abdominal ganglia, and the spike activity of PS motor neurons of the third (AG3) abdominal ganglion recorded under normal saline. The bathing solution outside the wells around the nerve chain was first washed out three times with 8 μM carbachol-containing saline and the spike activity of the AG3 PS motor neurons recorded 10 min after the third replacement of the carbachol-containing saline for 5 min. Subsequently the physiological saline inside the well of one ganglion was replaced with 5 mM L-arginine or 5 mM L-NAME-containing saline three times and the spike activity of the AG3 PS motor neurons recorded 10 min after the third replacement of the tested drug-containing saline for 5 min. Finally, the physiological saline inside the well of the other ganglion was replaced with L-NAME or L-arginine-containing saline and the spike activity of the AG3 PS motor neurons recorded 10 min after the third replacement of the second tested drug-containing saline for 5 min. Following the protocol above the PS motor neurons were recorded under four combinations of drug applications into AG4 and AG5 were performed.

For the third series of experiments (Fig. 5), petroleum-jelly wells were constructed around each of AG3 and AG4 abdominal ganglia, and the spike activity of AG5 PS motor neurons recorded. For the fourth series of experiments (Fig. 6), wells were constructed around each of AG3 and AG5 abdominal ganglia, and the spike activity of AG4 PS motor neurons recorded. The procedures used for drug application in the third and fourth series of experiments were similar to those of the second series.

### Analysis and statistics

All extracellular recordings were stored, displayed and analysed using a Power Lab (ADInstruments, Colorado Springs, USA). The cycle period of the rhythmic bursts of motor neuron spikes was defined as the time from the first spike of one burst to the first spike of next burst (Mita et al., 2014). The effect of drug application on cycle period was compared to the initial cycle period during the first carbachol application (control) and analysed using one-way RM ANOVA for multiple comparisons of treatments. Statistical analysis was based on the raw data but for clarity the data shown in the figures expressed as ‘relative cycle period’, and therefore with zero variance in the control group. Paired Student *t*-tests were occasionally performed to compare measured values. Cycle periods are expressed as mean±standard error of the mean (SEM) throughout. Statistical analyses were carried out using SigmaPlot v12.
